# ‘Wiener Null’ – levelling the city of Vienna

**DOI:** 10.1080/17535069.2018.1510025

**Published:** 2018-09-11

**Authors:** Angelika Psenner

**Affiliations:** Department of Urban Design, Vienna University of Technology, Vienna, Austria

**Keywords:** Vienna, Gründerzeit, topography, levelling, grading, inundation, traffic, building regulation

## Abstract

In 19^th^century Vienna was bounded by topographic elements of the hilly landscape and marsh land. Therefore,it could not easily spread and the regrading of the townscape was undertaken. Thus far, these aspects of historic urban planning have been relatively unexamined; this paper offers some essential findings.Grading adjustments occurred in the course of the two major city extensions or was triggered by infrastructural installation work. Further levelling was engaged—and organised by building codes—in order to improve traffic flow and for flood mitigation. The latter became the most effective intervention as it fundamentally restructured all low-lying districts.

## 1. Introduction – research background

Topography and spatial structures – the two main themes that fundamentally determine the city’s unique urban identity – which correlation do they have? Does the spatial structure follow the terrain relief that supports it, is it in harmony, in conformance with it or does the human being evolve its existential structure of life autonomously from the rational order of its mind? (Valena 1990, 8).

**Figure 1.**
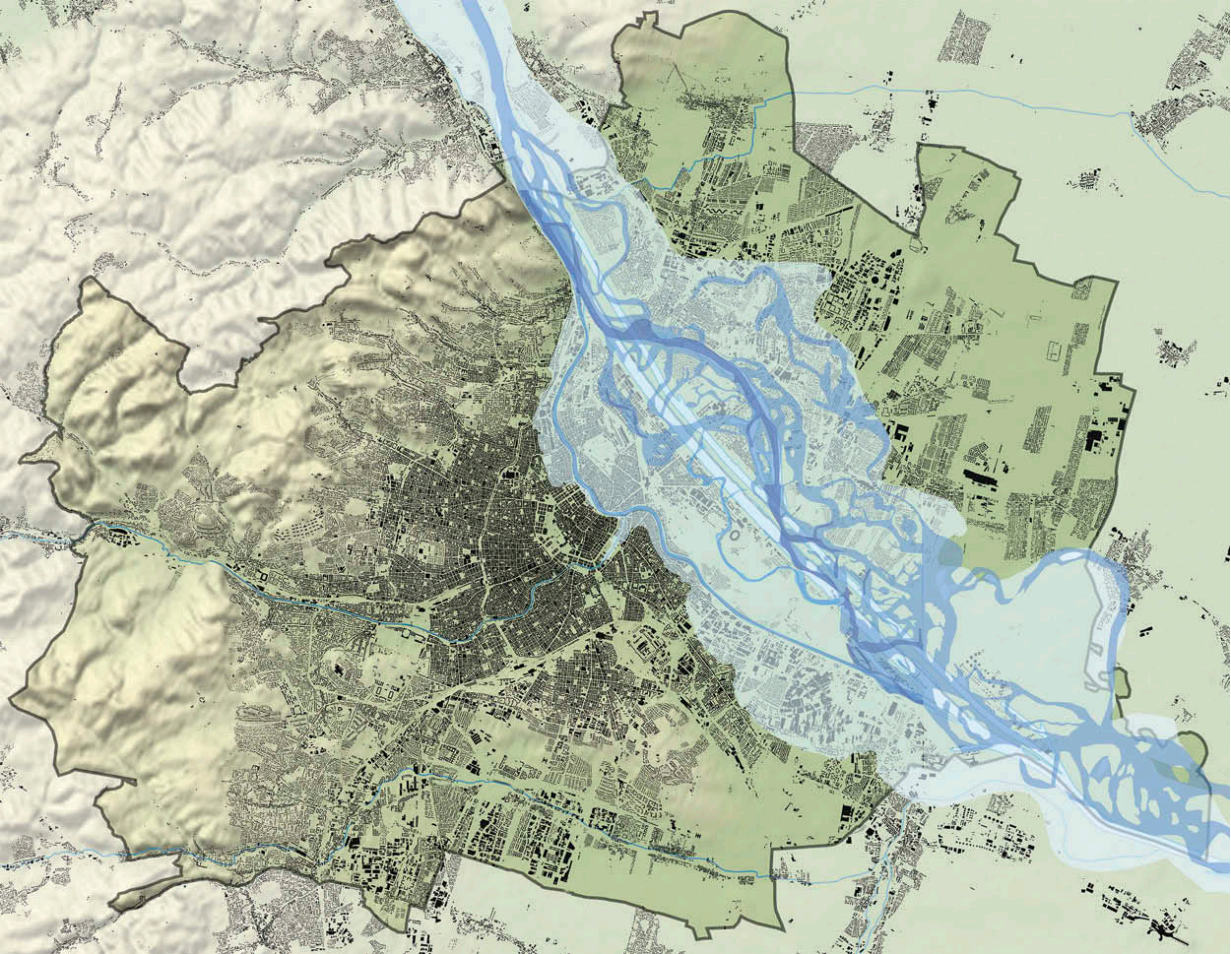
Plan showing the topography and the actual figure-ground-plan of the city, as well as the original waterways of the Danube and the districts affected by the great flood of 1830; © Kodydek/Psenner, TU Wien Sources: NordNordWest/Wikipedia, http://creativecommons.org/licenses/by-sa/3.0/de/legalcode; © OpenStreetMap- Mitwirkende (https://www.openstreetmap.org/copyright); https://www.schwarzplan.eu/produkt/lageplan-wien/); WStLA: Perspektivkarte des Erzherzogtum Österreich u.d.E.; Hohensinner 2015a, 13

Nestled between hilly landscape (the foothill of the Alps, Wiener Wald) and marsh land (of Danube River), the city of Vienna was prevented from easily spreading (see Figure 1). During Gründerzeit^1^ (1848–1918), when the city experienced a rapid population growth (from 400,000 to over 2.2 million inhabitants), the townscape was therefore modified radically: Not only has the river Danube and its wetland been regulated but also has the soil of greater parts of the city been levelled to a normative height. One aim of this large-scale intervention was to prevent slopes and to build straight and ideal streets that subsequently would form a grid that was ‘as evenly and uniformly as possible’ (BRC 1859 § 7 and BRC 1868 § 24); the other was to raise the city upon a certain ‘inundation’ level.

So far, this major change in the cityscape has not yet been investigated. This paper is therefore examining the precise scenario of this transformation and, thus, enlightening a peculiar and uncharted aspect of Vienna’s urban planning history.

### 1.1. State of the art

**Figure 2.**
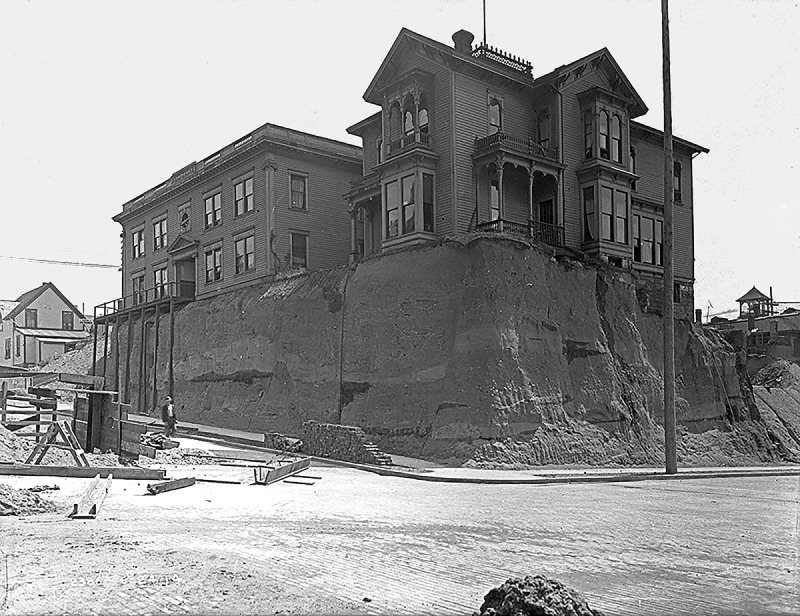
Seattle Regrade: a building in 1914, shortly before its demolishment and the regrading of the soil beneath, that used to be part of Danny Hill. Source: Seattle Municipal Archives. https://www.seattle.gov/Images/CityArchive/Exhibits/CityatWork/RossHotelRegrade.jpg

As mentioned, scientific literature does not cover the levelling topic, specifically in relation to Gründerzeit Vienna – Silvestru (2014) exclusively deals with the high medieval city structure in downtown. However, the levelling and flattening subject has been discussed with regard to other nineteenth century gridded cities, especially in the context to the implementation of the Commissioners Plan on Manhattan Island (Koeppel 2015; Ballon 2012; Yerkes 2011a, 2011b; Gandy 2002); the ‘Denny Hill Regrade’ in Seattle (Tarbill 1930; see Figure 2) or the ‘Seattle Underground’ intervention (Williams 2015; Seattle City Engineering Department 1979) and the ground level raise in Chicago (Wolf 2012; Cain 1972). Here the issue at stake was to, above all, describe the *engineering* process of the levelling; whereas Rose-Redwoods (2011) and Holloway (2013) also cover a cause analysis of the driving forces behind the endeavour. Scientific material can also be found on a more technical and methodological approach to the *levelling and grading of cities* theme, i.e. on the utilizing of new GIS technologies (Rose-Redwood and Li 2011), where again – from our perspective – the sociopolitical and cultural background are left out.

### 1.2. Methods – data

Our current research project ‘StadtParterre, Wien’ is exploring the urban parterre of the city of Vienna. A certain group of streets, all located in Gründerzeit quarters, are being examined and modelled in detail: thereby the structural state as well as the actual usage status of this urban system are being recorded in a so-called UPM^2^ (Urban Parterre Model, see Figure 3b). Beside the current UPM setting (see Figure 3c), a second 3D UPM (see Figure 3d) is being produced, by reconstructing the corresponding historical situation around 1910. The comparative analysis of the two models detects structural changes; furthermore, it reveals possible systemic interrelations and dependencies between structure and use of the individual StadtParterre elements (street, ground floor, basement and courtyard) (cf. Psenner forthcoming, 2017a, 2017b, 2015, 2014a) and the levelling and regrading of several lots and entire districts.

**Figure 3b.**
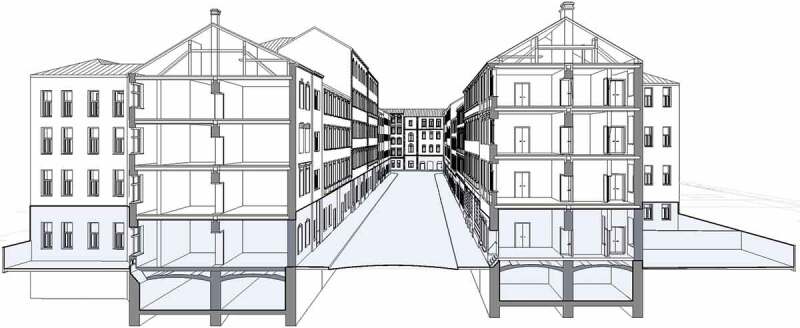
The UPM enlightens the StadtParterre concept: the urban parterre is a micro system, an interwoven texture of different zones: street, ground floor, basement and courtyard. The picture also shows the different level heights that were found in the research field and that lead to the here presented study. The name of the here depicted street had to be anonymised, as private data (building plans) was used in order to model the UPM. (© Kodydek/Psenner 2018)

**Figure 3c and 3d.**
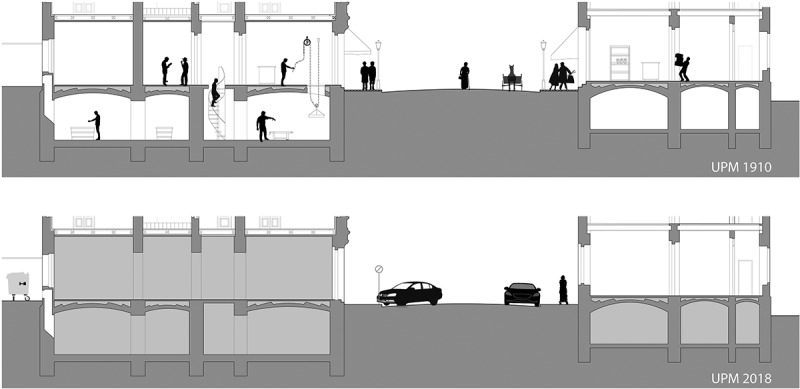
UPM sections (above: historic situation around 1910; below: current situation); amongst other things the microstructural approach of the UPM clearly shows the different height levels of courtyard, first floor, street that had been established during the urban levelling in the 19th century. (Vacancies and unused spaces are highlighted in grey) (© Kodydek/Psenner 2018)

We gather data directly from the field (through inspections, participatory observation and building surveys) and from various specific national and city archives^3^; these data are being viewed, photographed, analysed, evaluated and finally processed on architectural, sociological and historical terms (see Figure 3a). The most informative – to date and to this extend even the most untouched – data treasure is found in the building inspection authority archives. In addition to submission, and conversion plans, this data collection also includes business licences and permits for the usage of public (air)space (i.e. for setting up business signs and alike: so-called Platzzinsgenehmigung). This information allows for transcribing detailed ‘house biographies’ that cover structural and usage-related changes over the entire lifespan of the individual objects in the corresponding streets. The house biographies also detect the structure of changes in plot or street levels, whereas the microstructural UPM-approach shows the exact current level-situation in the relevant field: street, courtyard, cellar, souterrain, ground floor (see Figure 3c, Figure 3d and Figure 12).

**Figure 3a.**
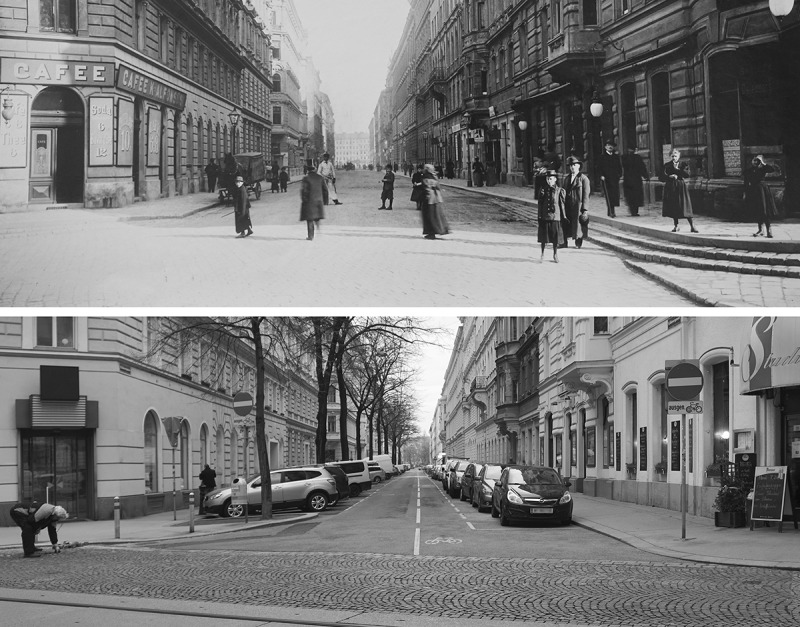
Glasergasse, above: around 1910 (source: Wien Museum – Inv.Nr. 203.019); below: photo of current situation (© Psenner 2018) By comparing the historic and the actual status of some exemplary areas the structural state, as well as of the usage of the different StadtParterre-zones is being thoroughly explored. The pictures also show a slight level-rise of the adjoining street (on the lower right corner of the pictures)

**Figure 12.**
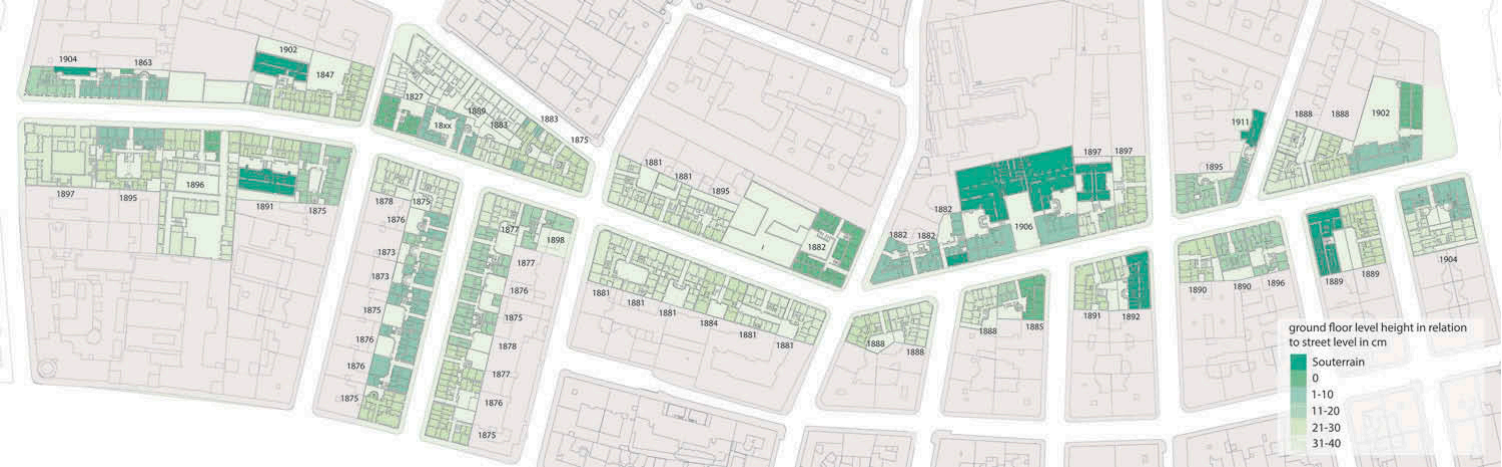
Level plan showing the consistently different parterre levels in Gründerzeit areas. The street stretch measures approximately two kilometres and is situated in one of the discussed areas, which have been affected by the level rise during Gründerzeit. Due to the used data (from urban planning archives) all information concerning the StadtParterre research project has to be anonymised. (source: UPM Urban Parterre Model © Kodydek/Psenner 2017)

**Figure 4.**
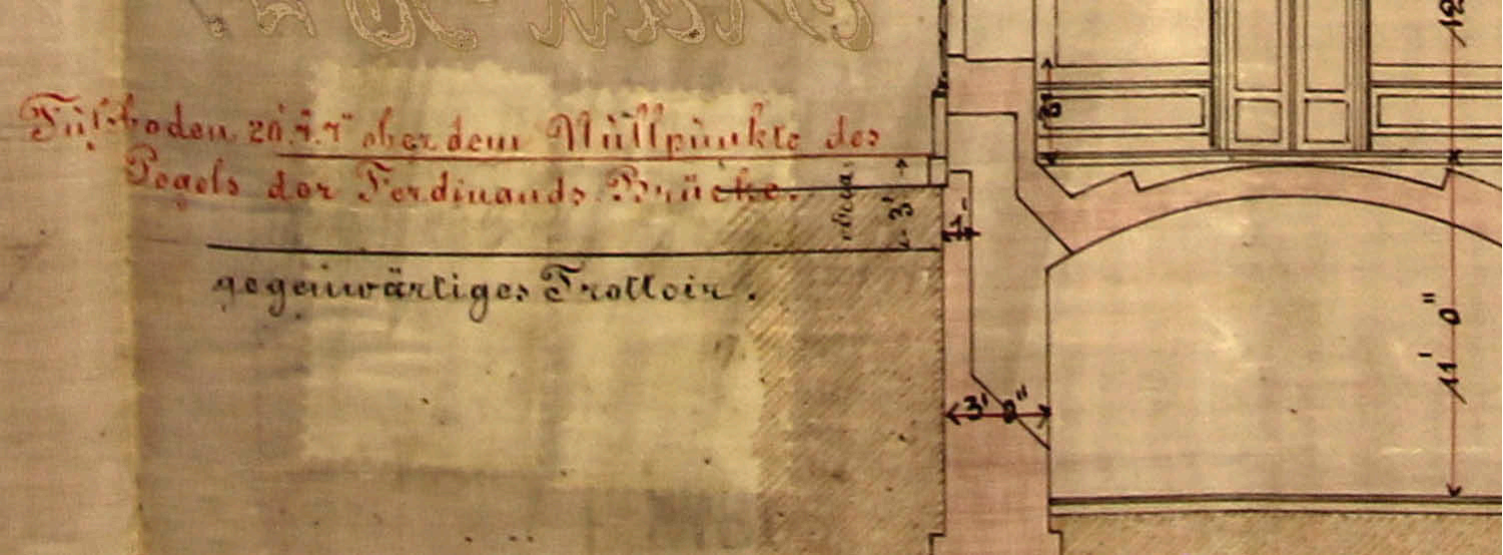
The picture shows the raising of the sidewalk-level by indicating the at the time height (“gegenwärtiges Trottoir”) and the new one, specified by city administration (without labelling); furthermore the level of the ground floor height is indicated (“Fußboden 20’ ober dem Nullpunkte des Pegels der Ferdinands Brücke”[ed. equals to Ferdinandbrücke, spelling from original source text]); (source: Psenner UPM ‘submission plan excerpt for a residential building with 3 floors in ... 1875’)

**Figure 5.**
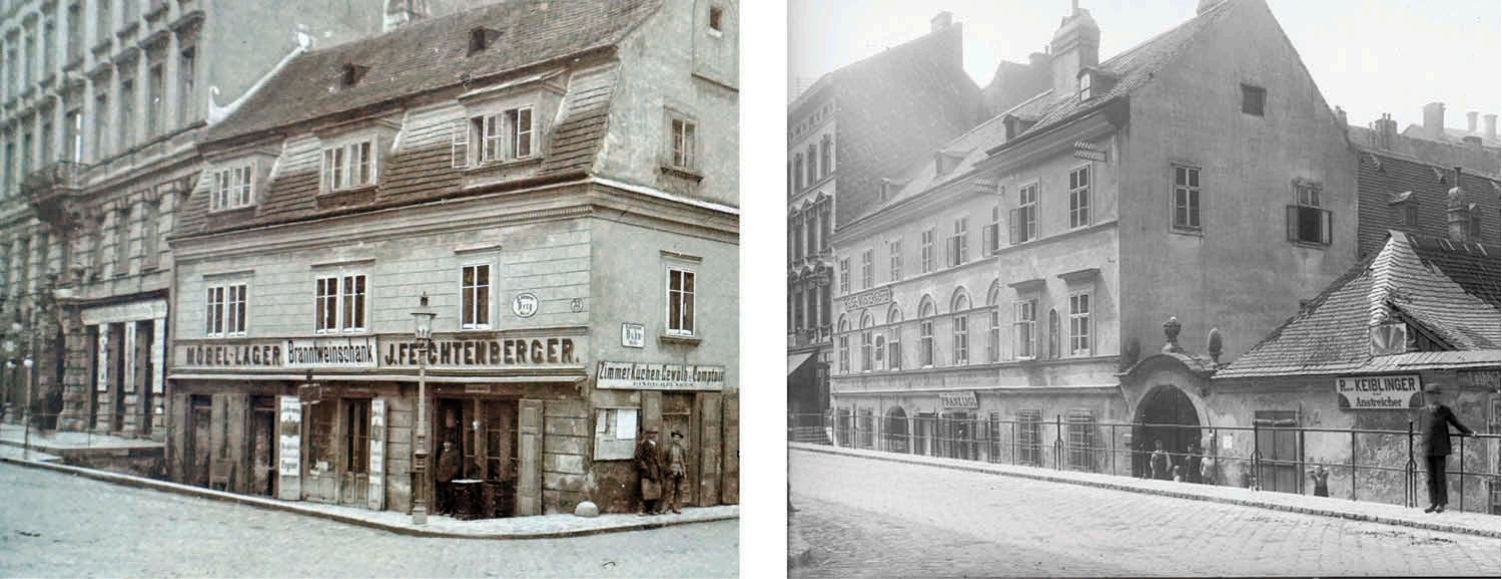
The pictures show how much the level of Hahngasse and Rotelöwengasse had been risen: while the Gründerzeit houses (on the left margin in both pictures) were at level with the new street, the older buildings had been banked up by more than one meter. Source: both pictures have been taken around 1902 by the famous Viennese photographer August Stauda; left: Hahngasse 7 (http://data.onb.ac.at/rec/baa1891324), right: Rotelöwengasse 5-7 (http://data.onb.ac.at/rec/baa1912938)

In the context of this work, we have now come across documents that show that the plot levels have been increased in the course of the urban development during Gründerzeit (see Figure 4) and that roads themselves have been raised considerably (see Figure 5). Since in scientific literature we found no concise explanation for this fact – the topic has apparently not yet found its way into the scientific debate – we had to get to the bottom of the matter, by conducting a side study, the results of which are presented here.

### 1.3. Research question

The main purpose of this side-research is to primarily *understand the background to the plot-related surface elevations found in historical submission plans in Vienna*. This precise focus (and the fact that this research aims to nourish the main StadtParterre-study) explains why certain aspects were given preferential treatment, such as the large-scale levelling of the low-lands, while others are mentioned for the sake of completeness but are not discussed in any detail. We hope that our text does stimulate a scientific perception of the – as yet untreated – aspect of the deliberate level rise of Gründerzeit Vienna in different scientific disciplines, such as urban history, architecture, urban planning research, urban research (sociology) and geodesy.

## 2. Findings

During the period in discussion (Gründerzeit), many changes – some quite considerable – were made in Vienna’s municipal area, concerning street and plot levels. By researching historical literature and planning documents, we located different motives for these alterations, which actually varied over time. This paper aims to explain and substantiate the various motives for an extremely elaborate and – given the technical possibilities during the nineteenth century – organization- and manpower-intensive intervention into the city landscape. During Gründerzeit, level regulations were effected in the course of

- the adjustment to the level of Ringstraße (around 1858 and afterwards)
- requirements from building codes (1829, 1859, 1868 and 1883)
  - to improve traffic flow
  - as a form of protection against floods
- requirements by the regulation master plan (after 1893)
- infrastructural installations: water supply and drainage (canalization, sewers), railway lines and railway beds, river restructuring etc.

**Figure 6.**
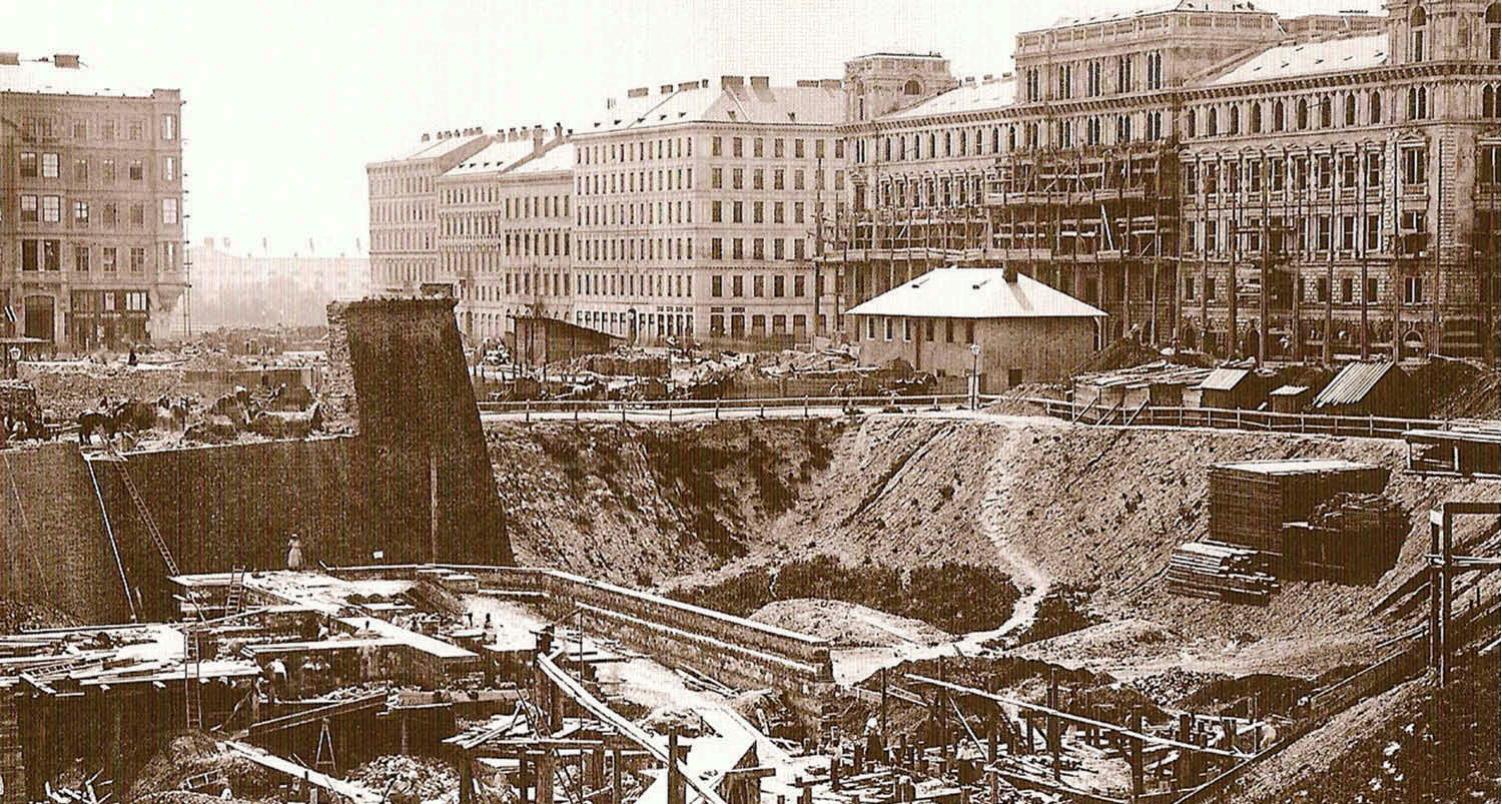
From the piece of medieval city fortification on the left side of the picture, one can deduct the original level height of the “glacis” and compare it to the much higher Ringstraßen-Niveau, which by 1863, when this photo was taken (author unknown) had already been leveled and built. („Founding of the opera“, source: catalogue of the exhibition: „Blickfänge einer Reise nach Wien - Fotografien 1860-1910 - Aus den Sammlungen des Wien Museums“; and: https://commons.wikimedia.org/wiki/File:Bau_Staatsoper.jpg

### 2.1. Level regulation in the course of the adjustment to the level of Ringstraße (around 1858 and afterwards)

After the first city expansion^,^^4^ and the following redesign of the Glacis area, a certain level was determined for the Ringstraße,^5^ which – compared to the original level – was distinctly higher (see Figure. 6). This rising of the terrain was at the time heavily criticized by practitioners and residents, as the surrounding streets and bridges had to be lifted considerably, in order *to improve conditions for passage*.^6^

No blame can be placed on the imperial bridge-building management, due to previous decisions made by the district council. Nevertheless, rather unpleasant results have occurred, from an aesthetic point of view as well as in view of passage conditions, which shall now be eliminated though raising the bridge [ed: Aspernbrücke]. The municipality should be charged with a fixed amount of the arising costs (6000 fl.), since it is responsible for the preceding level regulations. (N.N.: Wiener Zeitung, July 6th, 1864, 35)

When at a later point – in 1896 – Karl Mayreder,^7^ chief architect of the city regulation office, was entrusted with the urban planning for the first district, he stated: ‘Concerning the level regulations it is to be mentioned that (…) the existing level discrepancies would be alleviated everywhere as smoothly as possible’ (Mayreder 1896, 15–16).

**Figure 8.**
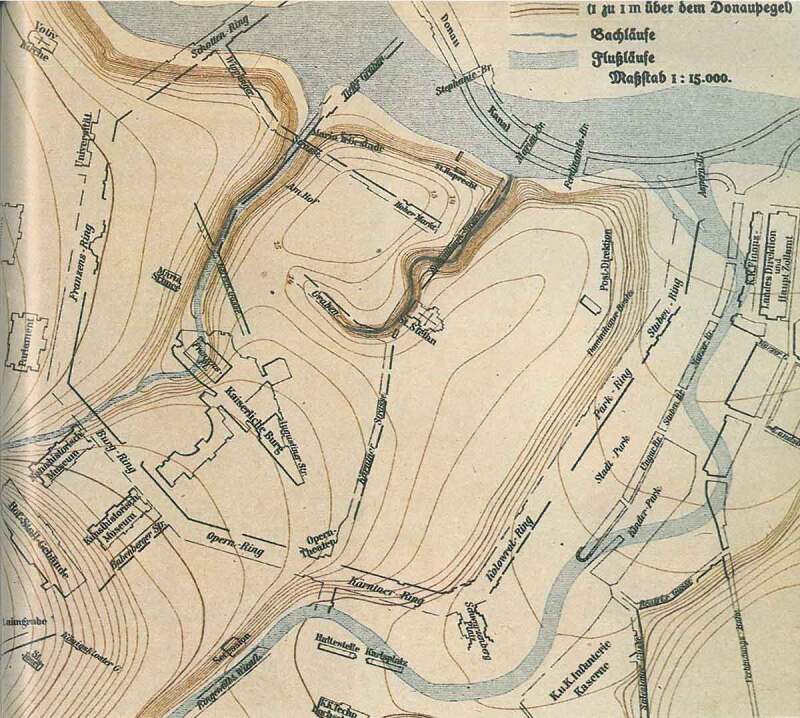
The pristine terrain relief plan, showing the first district and surroundings with the former waterways of Donaukanal, Wienfluss and Ottakringer Bach (St.-Ulrichs-Bach) - status before 1426 - and some of the later implementations (Ringstraße as planned and Donaukanal; status around 1857), source: Molik, Reining, Wurzer, 1980: 355

### 2.2. Level regulation in the course of requirements from building regulation codes (1829, 1859, 1868 and 1883)

**Figure 7.**
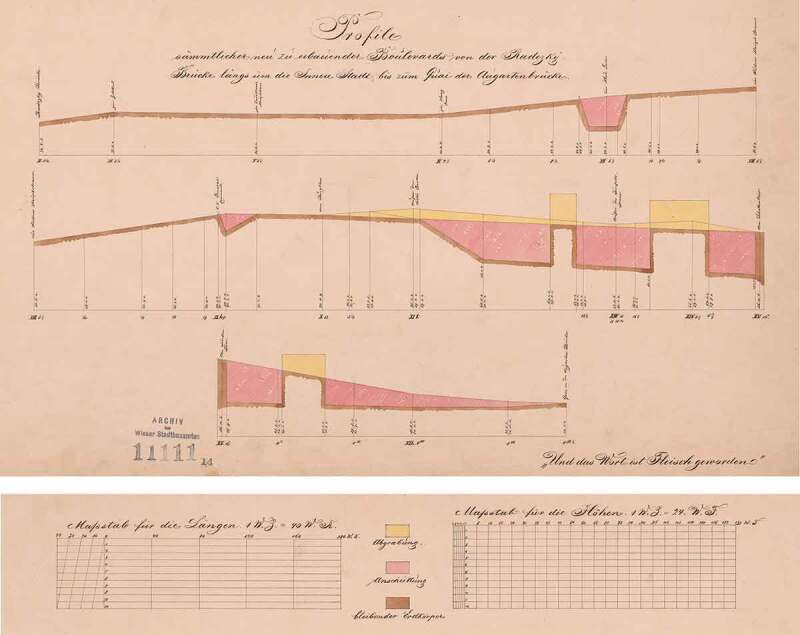
Entire hills were dug away, whilst building up valleys and lowlands in other parts of town. The plan shows the “profiles of all new boulevards” that were to be built according to Stache‘s competition entry for the city expansion in 1858: bright grey = excavation; grey: filled ground; dark grey: untouched earth (source: WAIS: Akt 3.2.2.P15.111111[26/1].14-20 - Concursprojekt Nr. 52; 1858)

The building code from the year 1859 was the first to stipulate urban planning guidelines for the construction of roads: they should be plotted *as straight as possible* (BRC 1859 § 7 cited in Förster 1859a, 338).^8^ In the 1868 building code, this provision was clarified as follows: ‘The authority has to ensure that streets are as straight and even and with as little level alterations as possible’ (BRC 1868 § 24 cited in Building Regulation Code 1868, 14). As a result, entire hills were dug away even in the first district, whilst building up valleys and lowlands in other parts of town (see Figure 7). The level regulation, aimed at by means of the new building code, had two goals: (a) the improvement of traffic flow and (b) the protection against floods – this will be discussed in detail in the following chapters.

**BRC 1883** (Building Regulation Code): When establishing the street and alley levels it has to be made sure that the streets and alleys are put into place with a gradient as low and balanced as possible and only under the utmost consideration of the existing conditions. (Building code for the imperial capital and royal seat Vienna. Law from 17 January 1883, Manz’sche Gesetz-Ausgabe, Vienna 1884, cf. Psenner 2005, 8)

#### 2.2.1. Level regulation to improve traffic flow

In the nineteenth century, traffic was increasingly seen as a driving force behind a *healthy, hygienic and flourishing city*. In his ‘Report of Motives for the Regulation Plan of the Inner City’, Mayreder writes: ‘An increase in real estate value and an improvement of sanitary conditions occurs mainly through the establishment of an extensive street network alone’ (Mayreder 1896, 20) (see: Figure 8). However, as early as 1859, Ludwig von Förster characteristically had titled his draft for Vienna’s city expansion with the words ‘The Straight Path is the Best’ (von Förster 1859b, 1).

The regulation of the inner city (…) has to accommodate the satisfaction of modern infrastructure needs, yet at the same time it must protect historically or artistically valuable buildings. Furthermore, since inner city traffic is concentrated in only a few main roads which have high real estate values, whilst right next to them are whole areas of empty, unhealthy and traffic-less streets which hold very low real estate values, special attention is to be paid to a traffic layout through such regions, which thereby not only gain in real estate value but are also remediated at the same time. (Mayreder 1896, 7)

**Figure 10.**
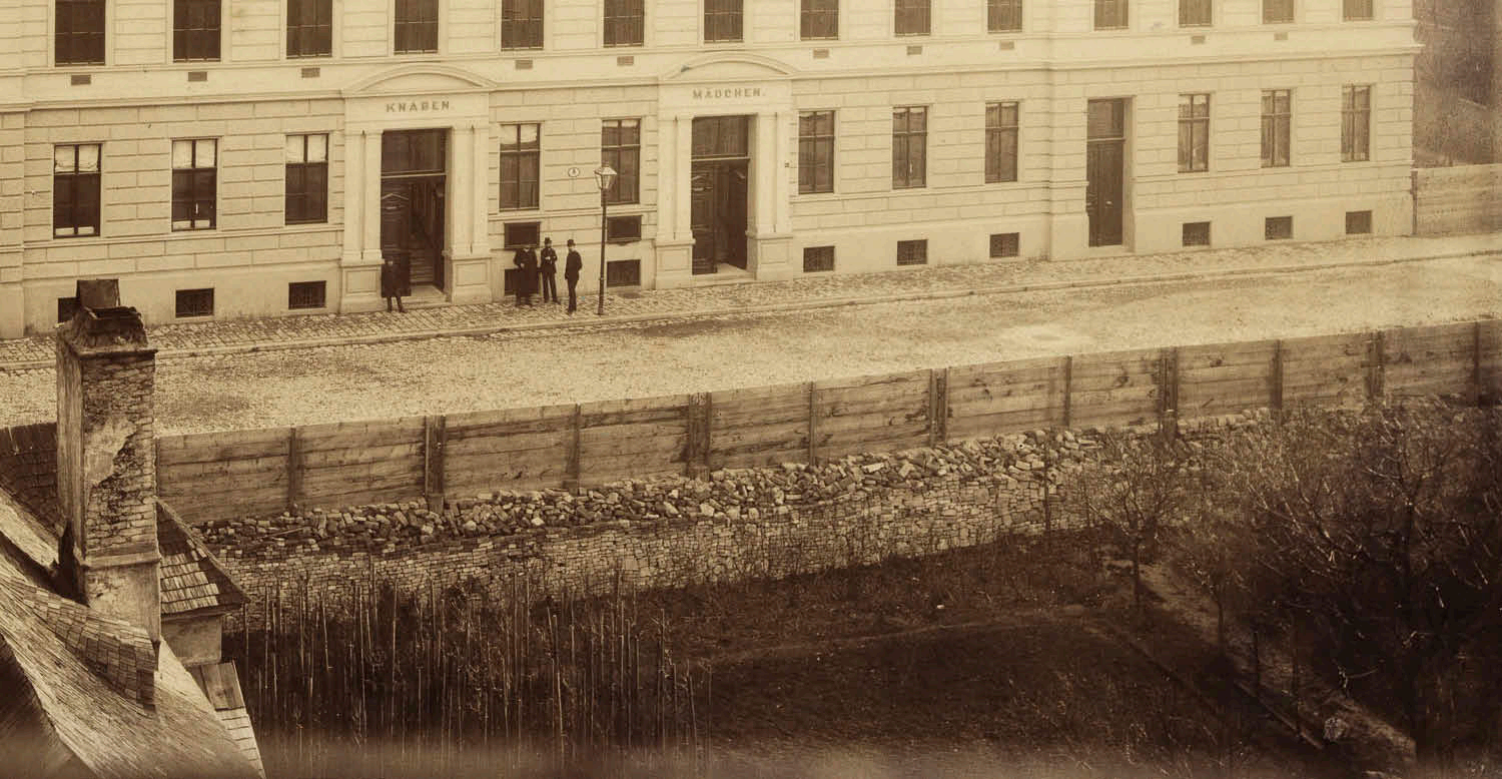
In Glasergasse the new Gründerzeit level height exceeds the original ground level by about 2 m. ‘IX. Glasergasse 8, Städtische Schule für Knaben und Mädchen’ (source: Wien Museum – Inv.Nr. 55.182)

Only a small number of the streets [located in the inner city] will retain a slope between 20 and 30‰ in the future, for example Wollzeile will have 27‰; the majority [will have a slope of] 20‰ or less. These remarks reveal that the planned road network will thoroughly meet the required road gradient ratios. (Mayreder 1896, 16)

One surely remembers, that the Quaistrasse is declining from Hotel Metropole to the entry of Rothenthurmstrasse, and from here on ascents once more. […] Due to raising this level […], the slope of Rothenthurmstrasse will be deteriorated in a favorable fashion. This change in ground level is rather significant, for at some sites a buildup of almost 2.5m will take place. (von Neumann 1887, 35)

Naturally, newly regulated streets often required a secondary level regulation of the adjacent plots of land:

The zoned building site [e.d. for the Rudolfs-Stiftung Hospital] was situated far below street level, thus a considerable mound of earth had to be heaped up to raise the hospital’s garden and courtyard terrain to the street level norm. (Förster and Ritter 1866, 2)

The fact, […] that the levelling which started at Fischerstiege requires a lowering of Salvatorgasse, which in turn results in the city hall being unearthed up to 2.3 m at the back, speaks in favour of a preservation of the former Bohemian Imperial Chancellery. (Mayreder 1896, 26)

The zoned building site for the new buildings [ed. Wilheminen-Spital] were partly situated fairly lower than the level of Flötzersteig, thus an extensive levelling became necessary, which was, for the most part, carried out through piling up earth. (Berger 1902, 71), [Franz Berger was the town planning director until 1908]

#### 2.2.2. Level regulations as a form of protection against floods

For a European city in the early nineteenth century, fire and flood disasters were the greatest environmental threats that had to be prevented from happening^9^ and as a matter of fact, the first building regulation code (erste Wiener Bauordnung), which was introduced by the government circular on 13.12.1829, characteristically derived from fire police bylaws (cf. Psenner 2013, 102–103).

In view of imminent flood risk, the building regulations, which were effective during *Gründerzeit*, specified, above all, new reference heights for each of the respective terrain levels, which had to be established. For along with the rapid growth of the city,^10^ the scale of damage as a result of floods grew as well; they reached a striking extent, especially in the city’s lowland (Hohensinner 2015a, 39–44).^11^ Hence, it is understandable that Vienna’s first countermeasure taken – which later on was accompanied by the channelization and structuring of creeks and streams – was the extensive levelling of the city. This intention was significantly supported by hygiene discourse reports published by the *Wiener Stadtphysikat*, ‘a forerunner of Vienna’s health department’ (Gierlinger 2015, 5) and other well-known physicians and scientists.^12^ Furthermore, urban landscape regrading obviously met the, at time, state of science, as other cities as well were taking this path – such as NYC Manhattan, Seattle and Chicago (Koeppel 2015; Williams 2015; Holloway 2013; Ballon 2012; Wolf 2012; Rose-Redwood 2011).

To date, this major project of city levelling concerted by Vienna during Gründerzeit has not been perceived as such. For the most part, drainage systems and the regulation and channelization of river courses – not least of all of the Danube itself – were the centre of attention (cf. Kortz 1905, 194–195; Hohensinner 2015a; Gierlinger 2015). However, even in 1900, the future city planning director, Heinrich Goldemund,^13^ writes

An important sanitary task fulfilled by the regulation was the systematic level raising of the city’s districts, which are situated next to the Danube, the Wienfluß and other wild creeks; which have often been struck greatly by devastating floods – frequently expanding across extensive sections of the city and subsequently ensuing poverty, misery and plagues. (Goldemund 1900, 14)

Whereas during the validity of the first building regulation code (from 1829), the new building level would be *individually* decided by a ‘building-assessment-commission’ (‘Bau-Augenscheins-Comission’) in dependence on *local conditions*, from **1859** onward it was necessary to gather the *official level standard* and disclose it on a special level-plan which was to be strictly implemented. In this way, a ‘systematic regulation of entire streets of houses’ (Goldemund 1900, 16) was now made possible. Henceforth, the determination of building lines and levels was the responsibility of the ‘building commission’ as first authority for reference (cf. Mayreder 1904, 70).

**BRC 1829**: In the suburbs, whose streets are not yet paved, the building-assessment-commission is obliged to deduce and establish, by all appearances, the level standard of newly built structures which is to be monitored, with regard to the local conditions and to pay particular attention to this, in many respects, profoundly important matter.

**Figure 13.**
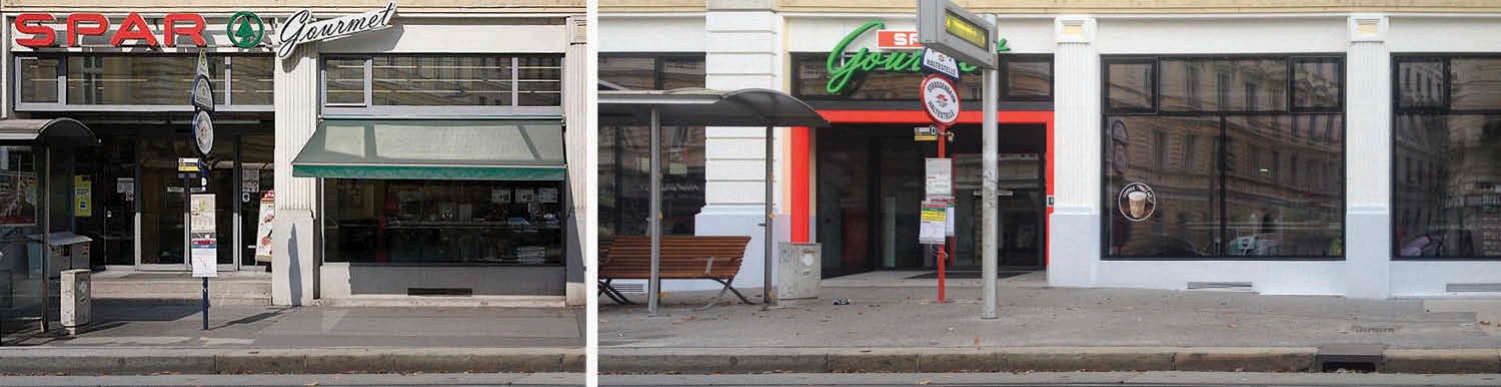
Pictures showing the entrance to a food store in Vienna, before (left) and after (right) the level-rise of the sidewalk in order to delete the entrance stairs and thus to ensure barrier-free access. (source: Psenner 2015, Psenner 2017)

The construction of new houses in the suburban areas, which are exposed to floods, will only be approved under the condition, that the entrance level [ed. ground floor] is raised adequately to local authorities’ direction and with regard to the water level during floods as well as consideration of the local conditions. The petition for raising must always be made apparent in the building plans. (Circular of the imperial and royal state government in the archduchy of Austria beneath the Enns, 1829, second section 2, § 22; as cited in Hagen 2015, 14)

**Circular 1838**: The level plan must show the alley’s gradient from the highest point to the outlet as well as the height levels of neighbouring entrances or driveways; for the suburbs which are exposed to the flood also the highest water level in the year 1830 has to be apparent.

For the local negotiation act these level plans are absolutely necessary in order to deduce from the observed existing level the new benchmark for ground floor and entrance level and to establish the needed lowering or raising [ed. of the building site] and the alley-paving according to § 22 of the building instruction code from 13 December 1829. (Circular of the imperial and royal state government in the archduchy of Austria beneath the Enns. 1831 as cited in Hagen 2015, 31)^14^

**BRC 1859**: § 1. With every new construction, extension or renovation carried out next to a public street, before applying for a regular construction permit, the building owner has to gather the official guideline for the building line and level and […] to present a proper situation and level plan.

§ 2. […] For the entire alley length, the indicated level – for the middle of the street as well as for the pavement on that street side, where the build shall be realized – must be clearly visible. This longitudinal level is to be set under a comparison line [Vergleichungslinie^15^], and has to include all relevant height differences […], as well as all entrance levels of the neighbouring buildings indicated in the plan. (Building Regulation 1859 section 1, as cited in Förster 1859a, 337)

**Figure 9.**
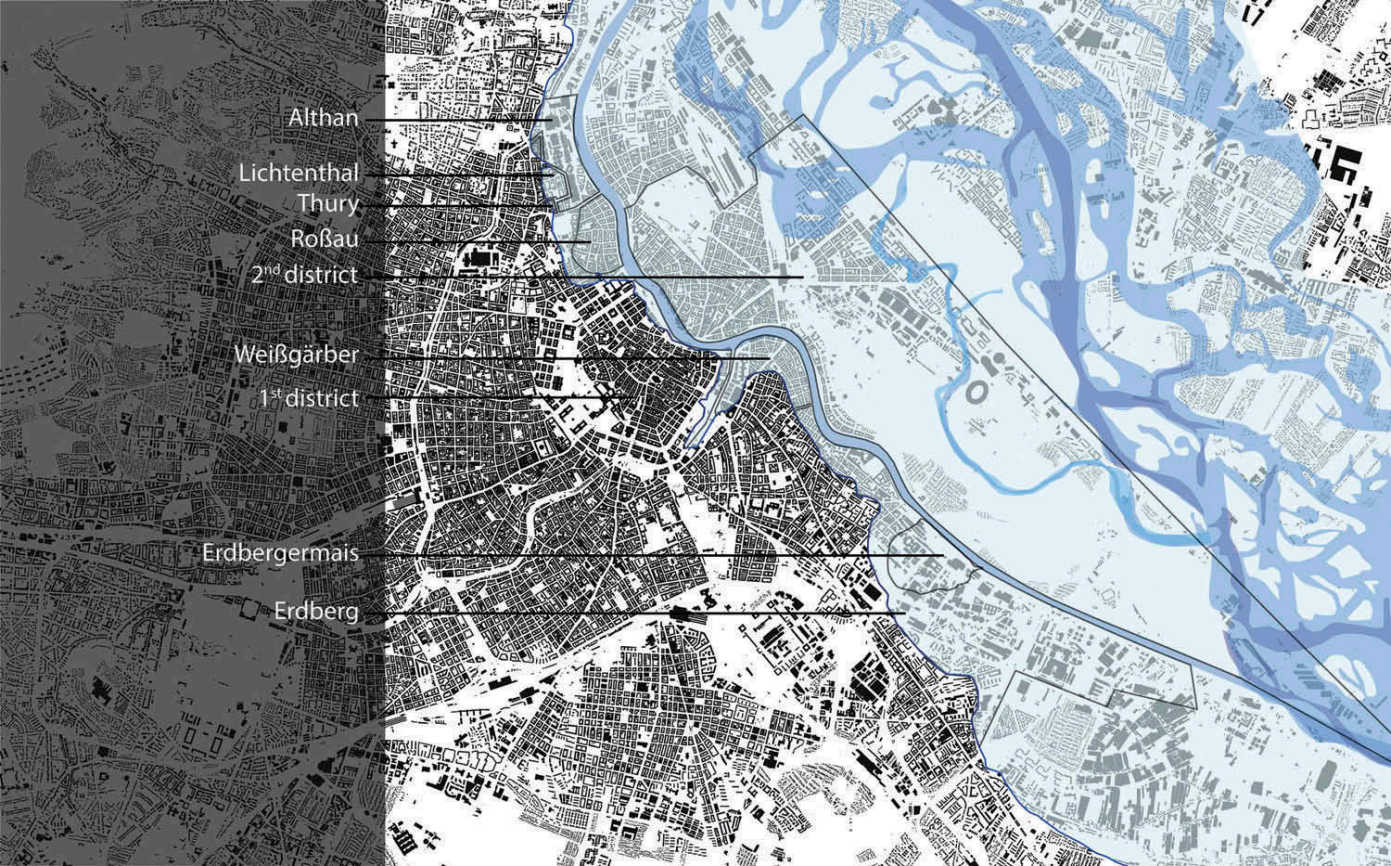
Plan showing the actual figure-ground-plan of the city, as well as the original waterways of the Danube and the districts affected by the great flood of 1830. As a matter of fact, this designated area is corresponding to the height-levelling zone and the quarters mentioned in the district council policy of 1882 when specifying the ‘flood area’: 2nd district, Erdbergermais, 1st district (some areas, near Donaukanal), Erdberg, Weißgärber, Roßau, Lichtental, Thury and Althan. © Kodydek/Psenner, TU Wien (Sources: https://www.schwarzplan.eu/produkt/lageplan-wien/; WStLA: Perspektivkarte des Erzherzogtum Österreich u.d.E.; Hohensinner 2015a, 13)

With regard to flooding-height parameters, the 1883 building regulation code set concrete guidelines: ‘in districts, which are exposed to a threat by floods, the level is to be decided according to the relevant flood regulations’ (Building Regulation 1883 § 2; L.G.Bl. Nr.35 as cited in Magistrat 1892, 4). In connection with the approval of *souterrain flats*, a special 1882 district council policy set precise guidelines in terms of ‘specification of the term “flood-area”’.^16^ Hence, the second district and the Erdbergermais (in the third district) especially, but also other ‘lower situated districts, respectively parts of districts’ were regarded as threatened by floods: ‘the areas of the 1^st^ district, which are situated in – or close to – the bank-level of Donaukanal, and […] the former suburbs Erdberg [ed. in 3rd district], Weißgärber [ed. in 3^rd^ district], Roßau, Lichtental, Thury und Althan [ed. all in the 9th district]’ (see Figure 9). Here, living in basement units (Souterrainlocalitäten) was allowed ‘only on a case-by-case basis with a special assessment of the municipal building control office and the medical office [ed. Stadtphysikat] and only until further notice’.^17^ A municipal regulation from the year 1889 finally controlled the use of basements as *work* space as well: ‘The legal usage of basement units as workshops does depend on the compliance with § 46 and with the commercial code. (Statth.-Erl. V. 29. Juni 1889, B. 13.086)’ (Magistrat 1892, 71).

Although by that time a unit measure had been established for the admissible underground level of souterrain flats, the handling of plot and street levels still varied from place to place. The ‘Instructions for Officials of the Municipal Planning Office for Application of the Building Law for Vienna from January 17, 1883’^18^ state the following:

ad § 1. **Request for official disclosure of the building lines and levels**. […] The specification for plot heights [levels to be newly created] has to be made with reference to the standard-zero water level at Ferdinandsbrücke [ed. equals to *Ferdinandbrücke*; old spelling from original source text]; also, are the fixed points – which are in proximity to the object under construction, and which are determined by the same standard-zero water level – to be declared. (pp 3–4)

ad § 2. **Special considerations for the determination of building lines and levels**. […] For the determination of the levels in districts exposed to a threat by floods, the following standards exist with respect to the main concept:

For Brigittenau the street level is determined as 14ʹ = 4.425 m above the local standard-zero water level of Donaucanal [equals to *Donaukanal*; here and in other source texts we decided to use the original old spelling].

In Leopoldstadt the level is to be maintained in those streets, in which the former required level of 18ʹ = 5.689 m above the local standard-zero water level is already established and the conditions allow this level to be retained, wherever else it is possible though […], the street level is to be lowered to 16ʹ = 5.057 m and in appropriate cases even to 14ʹ = 4.255 m above the local standard-zero water level.

With regards to level regulations in sections of the 3rd and 9th district which are situated in the inundation area, the same standards are valid as in the 2nd district. (pp 4–5)

The resolution taken by the local council in the year 1882, which is cited above, mentions a standardized reference height, namely ‘four meters […] above the local standard-zero water level in question’^19^ as a *municipality-wide applied standard level for the top edge of floors in basement flats* (Souterrain-Wohnungen).^20^ With this, therefore, in derivation of the preceding flood disasters, a uniformly – compared to the locally diverse level heights used beforehand – easier reference system was created. As all the level references before, it still related to the ‘*local* standard-zero water level’ as well and thus varied locally.

Here, one has to take into account that as a consequence of the great flood disasters during the nineteenth century (particularly those in the years 1830 and 1862), the Danube regulation was decided and carried out in the years 1870–1875. Because of the thereby obtained improvement, a lowering of the reference heights (from a former 5.85 and 5.25 m and similar, down to 4 m) followed. Yet, in 1897 and 1899, floods resulted in extensive damages once again. So, only after the regulation of the Donaukanal, a fix *reference height* could be set throughout the whole municipal area: ‘Only through the regulation and transformation of Donaukanal into a commerce and winter harbour, which was decided by law on 18 July 1892, was a fixation of the water level height in Donaukanal and the corresponding development of collective ducts made possible’ (Kortz 1905, 196). Subsequently, the standard-zero water level of the Donaukanal gauge at the Ferdinandsbrücke (historical zero point = 156.68 m above Adria (maA)) was determined as the *Wiener Null* and thus finally suited as a stable height reference for the whole municipal area: ‘The zero point of gauge at the Ferdinandsbrücke […] represents the reference mark to which all levelling stipulations on behalf of the city building authority refer’ (Swarowsky 1905, 5).^21^

### 2.3. Level regulation in course of requirements by the regulation master plan (after 1893)

The urban development plan (*Generalregulierungsplan*) of 1893 was established with the aim to manage all further planning interventions under inclusion of the above discussed level regulations.^22^

Even in 1864, the K.K. General-Kriegs-Commisär (royal and imperial General-War-Commissioner) V. Streffleur had demanded a universally valid regulation plan for the entire city in his paper ‘Vienna up to now and its Prospective Development’:

The greatest disaster and obstacle for the implementation of any idea conceived from a general perspective, lies in the lack of a universal plan that manages Vienna’s future development and in the powers held by public authorities, which are authorized to take individual decisions, depending on their own respective needs. (Von Streffleur 1864, 18)

Concerning this, Vienna’s planning director Goldemund writes retrospectively in 1900:

Due to regulations which state, that those parts of the city situated in the inundation area of the Danube have to be raised to at least 4.425 m above the local zero water gauge, and that the construction of basement flats is restricted to areas and streets that are safe from floods as a result of their level height, a thorough improvement of sanitary conditions was achieved in combination with the systematic regulation of the Danube and Wienfluss. (Goldemund 1900, 14) (see Figure 10)

German urban planner Josef Stübben also expresses his opinion concerning the relevant issue of this levelling choice in his work ‘Urban Development Hygiene’: Stübben detects *most grave sanitary disadvantages* through floods; for the establishment of new towns and districts, he therefore recommends:

if properties, which are exposed to floods, are to be developed, an artificial raising of streets and plots is to be arranged, so that not only the ground floor but also the basement rooms are above the river and ground water’s highest ordinate. If this is not possible due to financial reasons, at least the street level should be entirely free of water […]. (Stübben cited in: Hagen and Hauer 2015, 33)

### 2.4. Level regulation in the course of infrastructural installations: water supply and drainage (canalization, sewers), railway lines and railway beds, river restructuring etc

As mentioned above, most of the secondary scientific literature on the discussed matter can be found on the canalization-subject: Hauer, Hohensinner and Spitzbart-Glasl (2016); Hohensinner and Jungwirth (2016); Winiwarter et al. (2016); Hagen and Hauer (2015); Nierhaus and Wien Museum (2015); Czeike et al. (1981-2015); Gantner (2008); Wehdorn (1979); Bobek and Liechtenberger (1978); Stadler (19600. And also, original texts are often dealing with the canalization-approach Kohl (1905); Mayreder, Mayreder and Mayreder (1893); N.N. (1858, 1859); Sarau (1812); Schiefer (1856) or they are simply mentioning that due to some logistical matters, the street level or the plot level had to be raised.

Our town-planning department has […] applied for raising the ground level at the entry of Rothenthurmstrasse. Through raising the level, two purposes are served. Firstly, when building the Stadtbahn […] the new elevated street can cover the railway line, as well as make its construction easier…. (von Neumann 1887, 35)

Actually here, our text cannot essentially contribute to this highly sophisticated discussion; but as a matter of fact, we want to input a rather new aspect, which at the same time brings in the needed connex to the actual urban situation.

So-called Liechtental, a neighbourhood in the ninth district, was – and actually still is – lowland on especially low level, and therefore in nineteenth century hit by floods very often. Even after the river-regulation of the Danube, the flooding threat remained existent. The biggest problems here were heavy rainfall and cloudburst scenarios as occurred for example in 1 June 1898 and again in 17 July 1907, when the Alsbach flooded most of the surrounding plots and many people died (Gantner 2008, 113–114).

In consequence in 1898, the city-planning department decided to rebuild and expand the existing drainage system to prevent backwater. As this area was receiving large water volumes from many different rivers and creeks, all running down the Wienerwald hills, the then existing sewer profile dimension obviously had been insufficient. So that in case of heavy rainfall, drains, instead of absorbing water, would work like fountains: spitting out large quantities of water (Gantner 2008, 115).

**Figure 11.**
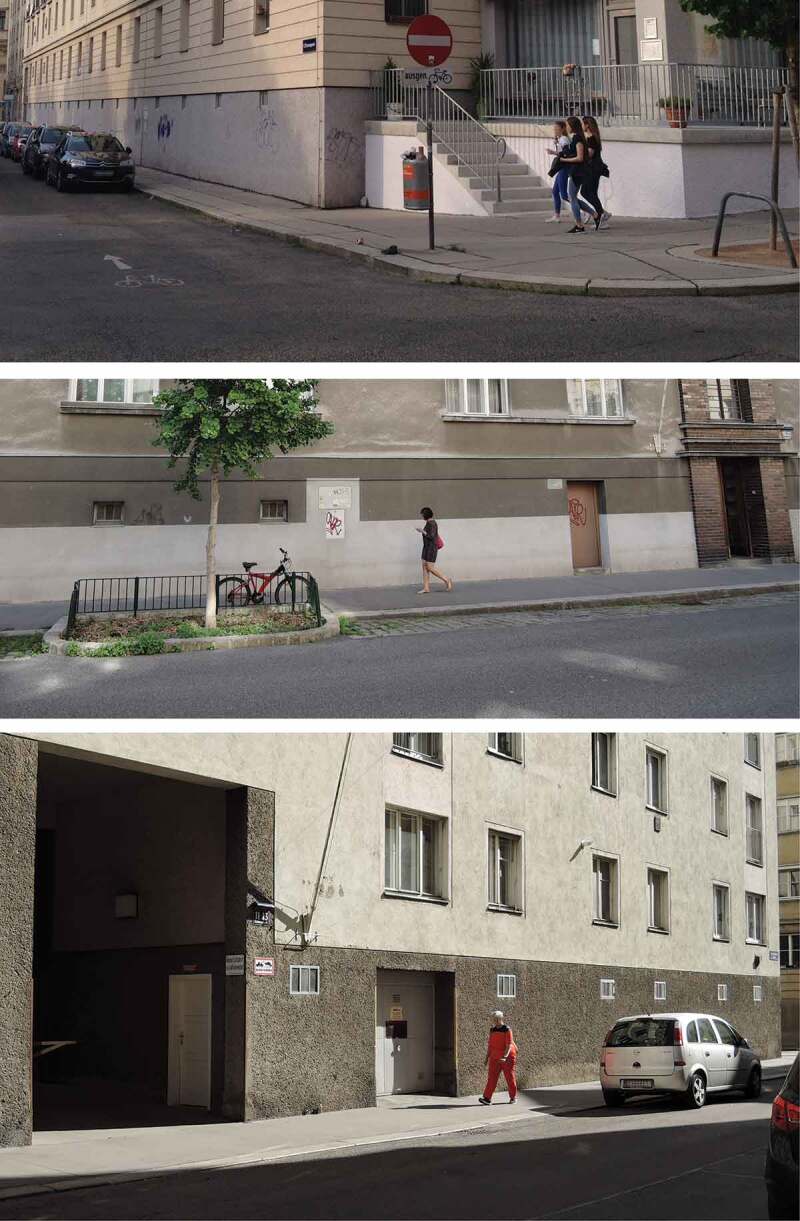
Houses in Liechtental without proper ground floors but cellars at ground level instead (© Psenner 2017)

Since then, many discharge and bypass channels have been built (Währinger-Bach- and Alsbachentlastungskanal; and plural rain spillover stations; cf Stadler 1960, 35 and https://www.wien.gv.at/umwelt/kanal/kanalnetz/#donaukanal); but at that time, when building new infrastructural installations was expensive and difficult to fulfil – as it meant to tear up the streets of whole neighbourhoods – and when WWI, WWII and the financial crisis delayed urban redevelopment, the city planning department again decided to slowly rise the street and plot level of the area: ‘The progressing urban development brought about a further levelling, when the lowest districts were lifted out of sanitary considerations – an endeavor which is still continued in the 9^th^ district’ (Swarowsky 1905, 5). Consequently, over a period of seven decades (from 1898 until around 1970), new building constructions in this area had to meet a so-called provisional street and plot level (‘provisorische Höhenlage’), thus anticipating a *future* higher street level (cf. Psenner 2008). This is why ground floors here were built with closed façades and tiny basement windows laying high up in the wall (see Figure 11).

In the end, the rising of the *streets* was not executed, rather did the term ‘provisorische Höhenlage’ completely disappear from all corresponding building plans submitted after 1970 (Psenner 2008). At the moment, we are trying to find prove for an informal statement which would feature the Catholic Church to have counteracted to the regulation, because the neglecting of the representative entry plinths and the burying of the entrance stairs of their churches (which, as a matter of fact, has happened elsewhere; i.e. at the nearby church Mariä Verkündigung in Servitenviertel, ninth district) had not found approval.

In any case, by simply repealing an, over decades highly effective level-regulation, Liechtental now consists of a considerable number of houses built between 1898 and 1970 that have cellars instead of ground floors: Among those e.g. Marktgasse 45 (built 1926–1927); here the ‘future new street level’ was indicated with *2.27 m above the original level*, so that beside a later ground level and cellar also a so-called ‘Tiefenerdgeschoß’, a ‘deep cellar’ was planned and built.^23^ In contrast to Seattle, however, this grading-transformation was never carried out so that ‘Vienna Underground’ (comp. to ‘Seattle Underground’ and the areaways described in Williams 2015) in this case lies *above* ground. And clearly those ground floors nowadays cannot be used for shops, restaurants, offices and alike, a situation which leads to the heavily discussed *StadtParterre* concept and to our actual research project (Psenner forthcoming, 2017a; 2017b).

### 2.5. Impact of historic level regulations

At the moment, still very few records can be cited concerning the logistical, technical and financial efforts that accompanied historic level regulations and its after effects. But from reading between the lines of some performance reports, we understand that a big effort indeed was invested in the laborious levelling endeavour – which Mayreder, in reporting on the ‘Regulierung der Innern Stadt’ (the urban redevelopment of the first district and especially the area next to Donaukanal), called *the most relevant changes from that time*; by this meaning the expansion of the road network together with the *extensive level increase at Salzgries (1879) and at Franz Josefs-Kai (1885)* (Mayreder 1904, 66–67).

The city expansion commission has resolved this task [Demolition of the most important objects in the Glacis area] within six years (1858 to 1864). The city walls are now completely demolished and the embankment remains only in places […] more than 100.000 cubic fathoms [682.100 m^3^]^24^ of soil were moved, in order to carry out this endeavour. (N.N. 1865, Wiener Kommunal-Kalender und städt. Jahrbuch, 125)

Furthermore, the project for the levelling, in so called Wasserleitungsstraße, in the 12^th^ district (to the right of Philadelphia-Brücke) was authorized […]. At the same time, the city council granted the required expenses of 123.125 K. The city council has likewise authorized the project for levelling at the extended Längenfeldgasse and at the access road to the new sections of Meidling cemetery in the 12th district and granted the expenses which amount to 188.000 K.^25^ (N.N 1907a, Der Bautechniker, 737)

The logistic behind the earth moving business has yet to be studied; so far, we did not find clues to certain storage locations or whether the soil had been drowned into water (i.e. the Danube) like in Seattle where much of the displaced earth from Denny Hill had been dumped into Elliot Bay (Tabril 1930, 487). Or – in the case when the level was raised – where the material had been taken from. Certainly, one has to bear in mind that all material had to be carried by small two-wheeled heavy load carts, so-called Khabskutschen.

In this context, however, historic newspaper articles like Rous’ publication in ‘Der Bautechniker’ from 1907 which criticises the fact of loss in value of houses which had been negatively affected by the levelling in the first and third districts (Rous 1907) are most instructive.^26^ Just like Neumann’s article which is discussing the same issue in the context of the realization of the Franz-Josefs-Kai elevation in the first district near Donaukanal: ‘If the realization takes place […], several individual older buildings will be buried extensively and lose one floor’ (von Neumann 1887, 35).

Secondary literature like Bobek and Lichtenberger’s book on Vienna’s’ urban development since the mid nineteenth century (originally dating from 1966) also can provide insight on the levelling-dependent changes in the former ‘Vororte’ like Hernals. But here certainly, further research is needed.

Hernals‘ old town centre was – simultaneously to the canalization of the Alsbach and in light of the loss in value of Biedermeier houses as a result of level regulations – entirely redesigned during Gründerzeit. [emphasis added] (Bobek and Lichtenberger 1978, 286).

## 3. Conclusion

The beginnings of nominal spatial planning in Vienna are commonly marked by the second great city expansion in 1890 and the implementation of the zoning plan (Bauzonenplan) and the Generalregulierungsplan in the year of 1893 (cf. Hagen 2015, 41). Yet, earlier than that – basically at the beginning of Gründerzeit – city administration co-ordinately and efficiently worked towards an overall structural design for the city. The fundamentally influential regulation of the topographical levelling deserves special attention when discussing this subject. The grading was laid out broadly, regardless of the administrative policy coordination and competence issues – which were due to the disconnected district areas (before the two great Stadterweiterungen – city expansions). The success was probably borne down by financial outsourcing: basically, individual land owners were responsible for establishing the ‘correct level’ on their plots. Only in a second phase, with the finalization of the street and pavement levels, the city managed the final readjustments.

This finding on the levelling sequence finally also explains the consistently different parterre levels in Gründerzeit areas: an urban development area tendered for a level increase was first allocated in plots; the single plots were then built on – not in running order, but – according to the possibilities of the new plot owners. As described in the text, the height specifications changed over the years, respectively, the developing of the plots followed different specifications. This, in the end, led to street stretches with unregulated courtyard and parterre levels with premise entrances on alternating heights (see Figure 12), whereas the building entrance levels (then often used as drive through) almost always were adapted to the street level.

Interestingly enough, given the current regulations on handicapped accessible buildings, it is precisely these different access levels that lead to a new levelling on a small scale: Businesses are now starting to raise the level of the sidewalks in order to ensure barrier-free access to their premises (see Figure 13).

## Notes

1. Originating from economic history, the term **Gründerzeit** actually refers to a period of promoterism in Central Europe of the nineteenth century, which began with the broad industrialization and lasted until the so called Gründerkrach (stock market crash of 1873). The somewhat fuzzy expression ‘Gründerzeitstil’ equals to the – from an art-historical point of view – correct term **Historism** and as such mainly describes the drawing on older styles (style pluralism), a phenomenon that was widespread in the nineteenth and early twentieth centuries in art, design and architecture. In describing urban developments that beside mere questions of stile also cover economic, social and political backgrounds, the term ‘Gründerzeit’ has to be preferred. The Viennese Gründerzeit is timed with 1848–1918 (sometimes also 1840-1918).

2. UPM – the urban parterre modelling and mapping method: a fundamental, in-depth description of this elaborate urban planning research method is given in Psenner (forthcoming, 2017b, 2014a, 2014b). In order not to blur the levelling-topic-focus of the presented paper, the description concentrates on the actual research steps related to this part of the study.

3. The archives of the building inspection authority (Baupolizei MA37), Wien Museum, district museums, Stadt- und Landesarchiv, Austrian National Library, Town Hall Library.

4. In 1850, the incorporation of 34 so-called Vorstädte was decided and spatially realized with the demolition of the medieval fortification after 1857. During the Gründerzeit, this ‘first city expansion’ was followed by a second one in the years 1890-1892 (with the incorporation of 33 so-called Vororte) and two smaller ones in 1904 and 1910.

5. The famous Wiener Ringstraße is a representative boulevard of almost 45 m width that surrounds the historic inner city of Vienna. It was built in the nineteenth century after the medieval fortifications (the town wall and the open field of the glacis) had been erased. The Ringstraße lines up the most important public buildings, like the houses of parliament, the town hall, the university, the national library, the bourse, the opera, the Burgtheater and many more expressive Palais of public and private use (Nierhaus 2015).

6. In her book about the Ringstraße, Elisabeth Springer amongst others mentions the contemporary criticism of the excessively high level of Ringstraße (cf. Springer 1979, 240 – many thanks to Sándor Békési for this hint).

7. Karl Mayreder taught at the K.K. Technische Hochschule in Vienna: he was ordinary professor for antique architecture and established the field of urban planning in 1899, for which he received the official teaching assignment in 1904. He was chief architect for the municipal regulation office from 1894 to 1902.

8. During Gründerzeit, the following building regulations were established: 1859, 1868 (including an amendment to the metric system in the year 1870) and 1883. Before 1859, the very first building regulation of the year 1829 was, of course, still relevant (cf. Psenner 2013, 103, 104).

9. The fear of epidemics and disease certainly influenced the architectural development and considerably phrased the building regulation codes as well; but actually, only after Ludwig Creutzer (1856), Carl Haller (1864) and other physicians had published studies on the connection between housing conditions and public health (cf. Psenner 2012). Before, anti-epidemic measures consisted mainly in sanitation (hospitals, city parcelling and rigorous control) and a rigid border defence with quarantine stations, where incoming travellers were held for several weeks. This included 17 ‘Sanitätskordone’ along the monarchies’ frontier and additional cordons and so called ‘Kontumazanstalten’ just outside the city border (Weigl 2018).

10. During Gründerzeit, the city recorded an absolute increase of 1.8 million inhabitants, which corresponds to a continuous annual increase of 25,000 people over a period of seven decades (cf. Kohl 1905; 197; Statistische Monatsschrift 1912).

11. Two particularly extensive flood disasters occurred in the years 1830 and 1862.

12. For example, the physician Ludwig Creutzer was able to prove the correlation between cholera outbreaks and the ‘low-lying areas of the city’, in a widely noted article, which he published in 1856 in the journal of Vienna’s Medical Doctor’s Association. (cf. Winiwarter et al. 2016, 228).

13. Along with Karl Mayreder, Heinrich Goldemund was head of the regulation office, which had been founded as a department of the municipal planning office in 1894. From 1913 onwards, he was Vienna’s city planning director (cf. AZW Architektenlexikon: http://www.architektenlexikon.at).

14. Two years previously, a similar announcement was published by the state government of lower Austria: [Level plans are to be drawn up] for buildings situated in the suburbs threatened by flooding [ed. and individual alleys at risk], whereby the highest water level has to be made apparent. The level plans […] are required for being able to deduce and establish the respective level from the local commission as well as make it apparent in the building plans, as stated in the building regulation § 22. (k. k. Nieder-Oester. Landesregierung. Nr. 12469 II. 1836; as cited in Hagen 2015, 31) In other cities around the monarchy, similar level guidelines followed owing to floods: in Pest, the building regulation was changed due to a large flood disaster in 1838: ‘[…] the raising of the ground floor of at least six inches above the flood level from 1838’ (Magistrat der königl. Freistadt Pest 1839, 149).

15. The height of this ‘Vergleichungslinie’ is district dependent; furthermore, it is calculated according to exact specifications and in relation to the local situation *and* the Donaukanal.

16. District council policy of the imperial capital and royal seat Vienna during the plenary session on 15 September 1882, sub Z. 1208; (Magistrat 1892, 71-72).

17. The construction and development of apartments on the ground or basement levels were already meticulously dealt with in the building regulation from 1868: in flooding areas generally, no basement flats were intended, furthermore ‘the floors of all ground level flats […] normally when constructing new buildings, had to be at least six inches [18.8 cm] above the determined street level’ and in flooding areas, the floors had to be above flood level.

18. The relevant issue dates from 1889 and has been published by Vienna municipal authorities (Magistrat 1889).

19. The municipal planning office was informed about this by a decree from 24.9.1882. Source: ‘Mag.-Decr. V. 24 September 1882, Z. 248.592’ (Magistrat 1892, 71-72).

20. In the year of 1865, a higher reference height was required, namely about 5.85 m above standard-zero water level: the ‘highest known water level of the Danube, which is 18’6‘‘ [ed. 5.85 m] above the standard-zero water level in the Donaucanal (viz. above the local standard-zero water level)’ (Mag. Bdg. Z. 104791 v. J. 1865; as cited in Hagen 2015, 32).

21. Later in time, the zero point of gauge at the Ferdinandsbrücke (now Schwedenbrücke) was lowered by 4 m (current zero point = 152.68 maA). In order not to create confusion, the meanwhile commonly used, well-established Wiener Null reference was not changed. Consequently, nowadays, the Wiener Null still does refer to the *historical* zero point, so to form the currently used conventional addition-constant of 156.68 maA (152.68 + 4) (cf. Berger and Ehrendorfer 2011, 123).

22. ‘The Generalsanierungsplan, which had been implemented originally as a planning instrument for the entire municipal area, was never completed as such. He always consisted solely of the current regulation plan parts which were valid at that time’ (Hagen 2015, 55).

23. More buildings with cellars at street level are to be found in: Reznicekgasse 18-22 (1937-1940); Marktgasse 15–17 (1937-1938); Thurygasse 6 (1955-1956); Marktgasse 9–13 (1955-1956); Lichtentaler Gasse 11–13 (1958–1959); Simon-Denk-Gasse 4–6 (1960-1961); Marktgasse 19 (1963-1965); Wiesengasse 32 (1968-1969).

24. 1/2kubikklafter = 3410,496 m^3^; therefore: 30,000 KK = 204.630 m^3^; 100,000 KK = 682.100 m^3^ (cf. also Wehdorn 1979, 124).

25. Cost comparison: in 1907, the daily newspaper *Illustrierte Kronen Zeitung* did cost one Krone and 60 Heller (in monthly subscription with delivery service). In the same year, a municipal roadman as well as a street lamp guard made a day’s salary of one Krone and 20 Heller (in the first year of service, as from the second year one Krone and 80 Heller were paid (cf. Arbeiter-Zeitung, 20 February, N.N 1907b; 7 and Arbeiter-Zeitung Mai 4th 1907c; 9).

26. The English translation of the articles’ title wold be: ‘The Concept for Vienna’s new Building Regulation Code and the Impact of the Paragraphs 54 −57 on the Land Price’. Actually, the draft presented by the municipal authorities in 1895 never got beyond the stage of pre-consultation. Only in 1929 – after various bylaws in 1920, 1927 and 1928 – a reformulated building regulation code was launched (Landesgesetzblatt No. 11/1930).
